# Is There an Association between Advanced Paternal Age and Endophenotype Deficit Levels in Schizophrenia?

**DOI:** 10.1371/journal.pone.0088379

**Published:** 2014-02-11

**Authors:** Debby Tsuang, Michelle Esterberg, David Braff, Monica Calkins, Kristin Cadenhead, Dorcas Dobie, Robert Freedman, Michael F. Green, Tiffany Greenwood, Raquel Gur, Ruben Gur, William Horan, Laura C. Lazzeroni, Gregory A. Light, Steven P. Millard, Ann Olincy, Keith Nuechterlein, Larry Seidman, Larry Siever, Jeremy Silverman, William Stone, Joyce Sprock, Catherine Sugar, Neal Swerdlow, Ming Tsuang, Bruce Turetsky, Allen Radant

**Affiliations:** 1 VISN-20 Geriatric Research, Education, and Clinical Center, VA Puget Sound Health Care System, Seattle, Washington, United States of America; 2 Department of Psychiatry and Behavioral Sciences, University of Washington, Seattle, Washington, United States of America; 3 VISN-22 Mental Illness Research, Education, and Clinical Center, VA San Diego Healthcare System, San Diego, California, United States of America; 4 Department of Psychiatry, University of California San Diego, San Diego, California, United States of America; 5 Department of Psychiatry, University of Pennsylvania, Philadelphia, Pennsylvania, United States of America; 6 VISN-20 Mental Illness Research, Education, and Clinical Center, VA Puget Sound Health Care System, Seattle, Washington, United States of America; 7 Department of Psychiatry, University of Colorado Health Sciences Center, Denver, Colorado, United States of America; 8 Department of Psychiatry and Biobehavioral Sciences, Geffen School of Medicine at University of California Los Angeles, Los Angeles, California, United States of America; 9 VA Greater Los Angeles Health Care System, Los Angeles, California, United States of America; 10 Department of Psychiatry and Behavioral Sciences, Stanford University, Palo Alto, California, United States of America; 11 Massachusetts Mental Health Center Public Psychiatry Division of the Beth Israel Deaconess Medical Center, Harvard Medical School Department of Psychiatry, Boston, Massachusetts, United States of America; 12 Department of Psychiatry, Mount Sinai School of Medicine, New York, NY, United States of America; VISN-3 Mental Illness Research, Education, and Clinical Center, James J. Peters VA Medical Center, New York, New York, United States of America; University of Iowa Hospitals & Clinics, United States of America

## Abstract

The children of older fathers have increased risks of developing schizophrenia spectrum disorders, and among those who develop these disorders, those with older fathers present with more severe clinical symptoms. However, the influence of advanced paternal age on other important domains related to schizophrenia, such as quantitative endophenotype deficit levels, remains unknown. This study investigated the associations between paternal age and level of endophenotypic impairment in a well-characterized family-based sample from the Consortium on the Genetics of Schizophrenia (COGS). All families included at least one affected subject and one unaffected sibling. Subjects met criteria for schizophrenia (probands; n = 293) or were unaffected first-degree siblings of those probands (n = 382). Paternal age at the time of subjects’ birth was documented. Subjects completed a comprehensive clinical assessment and a battery of tests that measured 16 endophenotypes. After controlling for covariates, potential paternal age–endophenotype associations were analyzed using one model that included probands alone and a second model that included both probands and unaffected siblings. Endophenotype deficits in the Identical Pairs version of the 4-digit Continuous Performance Test and in the Penn Computerized Neurocognitive Battery verbal memory test showed significant associations with paternal age. However, after correcting for multiple comparisons, no endophenotype was significantly associated with paternal age. These findings suggest that factors other than advanced paternal age at birth may account for endophenotypic deficit levels in schizophrenia.

## Introduction

### Paternal age and risk of schizophrenia

The children of older fathers have an increased risk of developing a number of psychiatric disorders that include deficits in behavioral and cognitive functioning. Studies have demonstrated associations between advanced paternal age and epilepsy [1], bipolar disorder [2], [3], autism [4], [5], [6], [7], dyslexia [8], neurocognitive impairments [9], and deficits in social functioning [10]. The literature also suggests a relationship between patients’ advanced paternal age at birth and the development of schizophrenia spectrum disorders. Malaspina and colleagues [11] found that after paternal age at birth exceeded 25, the relative risk of schizophrenia for offspring increased with each five-year increment in paternal age at birth and that the greatest risk occurred for the offspring of the oldest paternal group: fathers age 45 and older. In that study, older paternal age at birth remained a significant predictor of schizophrenia risk even after controlling for maternal age. Dalman and Allebeck [12] showed that having a father age 45 or older at birth was associated with a doubled risk of developing schizophrenia compared to having a father age 20 to 24. Similarly, Byrne and colleagues [13] demonstrated a sex-specific relationship between advanced paternal age and schizophrenia, showing that the risk for schizophrenia increased for sons of fathers age 55 years and older and for daughters of fathers age 50 years and older. However, a recent meta-analysis did not find gender differences [4], [14], [15], [16], [17], [18], [19].

### Paternal age, severity of symptoms, and cognitive functioning in schizophrenia

Among those offspring who develop schizophrenia, advanced paternal age has also been associated with severity of clinical symptoms. Rosenfield and colleagues [20] found that patients with no family history of schizophrenia and a paternal age at birth of ≥ 35 showed more severe symptoms (higher total scores and higher positive, activation, and autistic preoccupation scores on the Positive and Negative Syndrome Scale [PANSS]) during a medication-free phase than patients with younger fathers.

Others have suggested that paternal age is also associated with cognitive functioning in schizophrenia. Using demographic and clinical data collected from subjects with schizophrenia or schizoaffective disorder, Lee and colleagues [21] defined paternal age–related schizophrenia (PARS) as having a father age 35 or older at the time of the subject’s birth and not having a family history of schizophrenia. They performed numerous *k*-means cluster analyses and found that 20 of 34 (59%) individuals with PARS clustered in a group which exhibited significantly greater discrepancy between verbal and performance intelligence on the WAIS-R subtests compared to subjects in six other clusters. These findings suggest that the cognitive performance deficits in patients with schizophrenia may be associated with advanced paternal age.

### Goals of the current study

Despite the evidence demonstrating paternal age effects on the development of schizophrenia-spectrum disorders in offspring, we do not know whether advanced paternal age is associated with the endophenotypic deficits observed in schizophrenia. By studying these associations, we have the opportunity to understand whether paternal age has differential effects on schizophrenia-related neurophysiological and neurocognitive endophenotypic deficits with known neurobiological functions. Endophenotypes, which were first described by Gottesman and Gould [22], are defined as stable traits that are more common in affected individuals but are not associated with the severity of the illness, that cosegregate with illness in families, and that are more common among family members of affected individuals.

Studying the impact of paternal age at birth on both individuals with schizophrenia and their unaffected siblings may highlight important relationships between nonfamilial factors and endophenotypes for schizophrenia. Thus, in the current study, we hypothesized that advanced paternal age would be associated with greater deficits in neurophysiological and neurocognitive schizophrenia-related endophenotypes.

## Materials and Methods

### Clinical ascertainment and assessment

The current study utilizes data from the Consortium on the Genetics of Schizophrenia (COGS) [23], , a multisite, family-based study on the genetics and heritability of neurocognitive and neurophysiologic schizophrenia endophenotypes. Participants in this study met COGS criteria as schizophrenia probands (n = 293) or unaffected siblings of those probands (n = 382). The probands and their unaffected siblings completed a structured clinical diagnostic interview (the Diagnostic Interview for Genetic Studies [26]) and a best-estimate consensus diagnostic procedure that included a comprehensive evaluation of psychotic, mood, and substance use disorders and related symptomology [23], [27]. This information was used in concert with a standard medical record review to establish handedness, age at onset of psychosis (for probands), education level, parental education level, and parental age; the Family Interview for Genetics Studies (FIGS) [28] was used to assess subjects’ family history of schizophrenia or schizoaffective disorder; and the reading subtest of the Wide Range Achievement Test, 3rd edition (WRAT-3) [29], was used to estimate premorbid intellectual functioning. The probands were also assessed using the Schedule for the Assessment of Negative Symptoms (SANS) [30] and Schedule for the Assessment of Positive Symptoms (SAPS) [31]; all probands met Diagnostic and Statistics Manual of Mental Disorders, 4th edition (DSM-IV), diagnostic criteria for schizophrenia. Recruitment and ascertainment strategies, in-depth inclusion and exclusion criteria, and in-person training and quality assurance guidelines for the assessment team and endophenotype testers are detailed in an earlier manuscript by Calkins and colleagues [23]. The COGS encompasses study sites at the University of California, San Diego; University of California, Los Angeles; University of Colorado, Denver; Harvard University; Mount Sinai School of Medicine; University of Pennsylvania; and University of Washington; institutional review boards at these sites and/or their affiliated institutions approved the COGS procedures in adults age 18 and older. All subjects demonstrated an understanding of their involvement in the study and provided signed informed consent before commencing study procedures as well as ongoing verbal and physical assent throughout the study.

### Endophenotype assessment battery

The COGS endophenotype assessment battery and its use in the different COGS studies have been discussed in detail elsewhere. For the current study, endophenotypes included standard assessments of antisaccade performance (proportion correct) [32]; prepulse inhibition (PPI) [33], which was measured as 100 × [1 − (magnitude of startle to pulse preceded by prepulse/magnitude of startle to pulse without a preceding prepulse)] using 60 msec pulses; the Degraded Stimulus (DS), 3-digit Identical Pairs (IP), and 4-digit IP versions of the Continuous Performance Test (CPT), which were measured using the signal/noise discrimination index (d′) [34]; the Forward and Reordered condition of the Letter-Number Span (LNS) [35], measured as the total number of correctly recalled sequences; the California Verbal Learning Test (CVLT), specifically the total and total semantic clustering scores on trials 1–5 (CVLT total and CVLT semantic) [36]; and the abstraction and mental flexibility, verbal memory, face memory, spatial memory, spatial processing, sensorimotor dexterity, and emotion processing cognitive domains of the Penn Computerized Neurocognitive Battery (CNB) [34], [37], which were measured using the number of correct responses standardized to a z-score based on the COGS community control subjects.

### Data analysis

Demographic differences between schizophrenia probands and their unaffected siblings were tested using linear mixed-effects models [38] for continuous variables and generalized linear mixed-effects models [39] for categorical variables. In these analyses, family membership served as a random effect to account for the relatedness of observations among family members. Families in which at least one parent or sibling of the proband also had a diagnosis of schizophrenia or schizoaffective disorder were considered multiplex; all other families were considered simplex. Paternal age at birth was calculated by subtracting offspring age from parental age; it was treated as continuous.

To assess a possible effect of paternal age on the severity of symptoms, or on the age of onset of symptoms, separate linear models were used to model SANS, SAPS, and age of onset as a function of paternal age, gender, and a paternal age–by-gender interaction.

We conducted two separate types of analyses to test the association of each endophenotype with paternal age: proband-only analyses and simultaneous analyses of both the probands and their unaffected siblings. Our proband-only analyses consisted of linear regression models in which an endophenotype was modeled as a function of paternal age along with the covariates test site, subject age, subject gender, multiplex status, and parental education (maximum grade level of mother or father), as well as paternal age–by-gender and paternal age–by–multiplex status interaction terms. Simultaneous analyses of probands and their unaffected siblings consisted of linear mixed-effects models that mirrored those used in the proband-only analyses but with family as a random effect, with group (proband versus sibling) as a fixed effect, and with additional interaction terms: all second-order interaction terms involving group. For all analyses of the CVLT endophenotype, WRAT-3 standardized reading scores were included as a covariate in place of parental education as suggested by Stone and colleagues [36]. Missing values for parental education and WRAT-3 scores were imputed as described previously [36], [40].

For any term involving paternal age at subject birth, nominal significance was defined as a *P* value less than 5%. Adjustment for multiple comparisons was performed using the method of Holm [41]. One set of adjustments accounted for the 16 endophenotypes, and another set of adjustments accounted for the 16 endophenotypes and two models (probands only versus probands plus unaffected siblings). Neither set of adjustments accounted for the presence of multiple terms that involved paternal age at birth.

We performed three sets of sensitivity analyses: one in which interaction terms were omitted, one in which paternal age was treated as categorical (<40 years old versus ≥40), and one in which interactions terms were omitted and paternal age was treated as categorical.

All analyses were performed using R version 2.12.2 [42]. Linear mixed-effects models were fit using the R package *nlme* [43], and generalized linear mixed-effects models were fit using the R package *lme4* [44].

## Results

### Demographic and clinical variables

For subjects with at least one valid endophenotype, paternal age at birth was obtained from 293 of 345 (85%) schizophrenia probands and from 382 of 456 (84%) unaffected siblings. The 382 unaffected siblings represent 279 families, of which 17 (6%) were multiplex; 18 of the 293 probands (6%) were from multiplex families. As expected, probands and siblings were similar in age and paternal age at birth, but probands had a significantly higher proportion of males and smokers, had significantly lower average education levels, and performed worse on the WRAT-3 (Table 1). Of the 293 probands, 23 (8%) were born to fathers 40 years old or older.

**Table 1:** Demographic and clinical characteristics of schizophrenia subjects and unaffected siblings.

	Schizophrenia subjects (N = 293)		Unaffected siblings (N = 382^a^)			
	n	%	n	%	*P* value^b^	Odds Ratio (95% CI)^c^
Gender (male)	222	76	173	45	<0.0001	3.9 (2.8, 5.5)
Race (white)	216	74	305	80		
Smoker^d^	126	44	62	16	<0.0001	4.0 (2.7, 5.7)
Multiplex status^e^	18	6	28	7		
						
	Mean	SD (Min, Max)	Mean	SD (Min, Max)	*P* value^b^	Difference (95% CI)^c^
Age (years)	33.7	10.5 (18, 62)	35.0	11.1 (18, 66)	0.21	
Education (years)^f^	13.6	2.1 (8, 20)	15.4	2.4 (7, 25)	<0.0001	−1.7 (−2.0, −1.4)
Parental education (years)^g^	15.9	3.4 (0, 25)	15.8	3.6 (0, 25)		
WRAT-3^h^	102.5	10.9 (69, 122)	106.5	9.5 (64, 125)	<0.0001	−4.0 (−5.3, −2.7)
Age at onset of symptoms (years)^i^	20.9	5.5 (6, 51)				
SANS^j^	9.5	6.0 (0, 25)				
SAPS^k^	6.2	4.1 (0, 20)				
Paternal age at birth (years)	30.7	6.3 (18, 63)	30.7	6.3 (16, 66)	0.36	

Abbreviations: CI, confidence interval; SANS, Schedule for the Assessment of Negative Symptoms; SAPS, Schedule for the Assessment of Positive Symptoms; SD, standard deviation; WRAT-3, Wide Range Achievement Test, 3rd edition.

a Unaffected siblings represent 279 families, of which 17 (6%) were multiplex.

b Between-group *P* values are based on generalized linear mixed-effects models for categorical variables and linear mixed-effects models for continuous variables; *P* values were not computed for race, multiplex status, parental education, or age at onset of symptoms.

c For continuous variables, differences between groups (probands – unaffected siblings) and 95% CIs for differences are based on linear mixed-effects models; for categorical variables, odds ratios and 95% CIs for odds ratios are based on generalized linear mixed-effects models. CIs are shown only for variables where the *P* value for the between-group test is <0.05.

d 4 missing values for schizophrenia subjects; 2 missing values for unaffected siblings.

e Multiplex families were those for which at least one parent or sibling of the proband had a history of schizophrenia or schizoaffective disorder.

f 2 missing values for schizophrenia subjects; 2 missing values for unaffected siblings.

g 6 missing values for schizophrenia subjects; 3 missing values for unaffected siblings.

h 10 missing values for schizophrenia subjects; 12 missing values for unaffected siblings.

i 5 missing values for schizophrenia subjects.

j 7 missing values for schizophrenia subjects; 73 missing values for unaffected siblings.

k 7 missing values for schizophrenia subjects; 74 missing values for unaffected siblings.

### Association of paternal age with symptom levels and age of onset in probands

Seven (2%) of the probands were missing values for SANS and SAPS, and 5 (2%) were missing values for age at onset; these probands were all born to fathers less than 40 years old. None of the linear models for SANS, SAPS, or age of onset in probands showed a significant effect for paternal age or a paternal age–by-gender interaction.

### Association of paternal age with endophenotypic deficits

Table 2 presents descriptive statistics for the endophenotypes by subject group. Table 3 displays the results of linear models that test the association of paternal age at birth with endophenotypic deficits in probands alone and the results of linear mixed-effects models that test the association of paternal age at birth with endophenotypic deficits in probands and unaffected siblings combined.

**Table 2:** Unadjusted endophenotypes for schizophrenia subjects and unaffected siblings.

		Schizophrenia subjects (N = 293)				Unaffected siblings (N = 382)		
Endophenotype		n	Mean	SD (Min, Max)		n	Mean	SD (Min, Max)
Antisaccade (proportion correct)^a^		242	0.62	0.26 (0, 0.98)		331	0.81	0.20 (0.02, 1)
PPI (percent)^b^		198	44	28 (−107, 90)		275	46	26 (−64, 94)
DS-CPT (d′)^c^		252	2.4	1.1 (0.1, 5.4)		349	2.7	1.0 (0.0, 5.3)
CPT-IP 3-digit (d′)^d^		242	2.2	0.8 (−0.2, 4.3)		348	3.0	0.8 (0.0, 4.8)
CPT-IP 4-digit (d′)^e^		241	1.3	0.7 (−0.2, 3.5)		348	2.0	0.8 (−0.3, 4.8)
LNS Forward (number correct)^f^		282	13	3 (4, 21)		370	14	3 (4, 21)
LNS Reordered (number correct)^g^		281	9	3 (1, 18)		368	11	3 (4, 20)
CVLT total (number correct)^h^		277	43	13 (11, 75)		369	55	9 (28, 76)
CVLT semantic (number correct)^i^		277	0.5	1.5 (−2.2, 7.6)		369	1.9	2.2 (−2.4, 8.4)
CNB (z-transformed number correct)^j^								
Abstraction/mental flexibility		258	−0.56	1.2 (−2.5, 1.3)	347		0.06	0.88 (−2.5, 1.3)
Verbal memory		149	−0.71	1.2 (−4.0, 1.5)	177		−0.03	0.93 (−4, 1.5
Face memory		267	−0.83	1.2 (−4.0, 1.6)	350		0.09	0.86 (−3.2, 1.7)
Spatial memory		261	−0.61	1.1 (−3.4, 1.8)	349		0.09	0.87 (−2.4, 1.8)
Spatial processing		247	−0.06	1.1 (−3.7, 1.4)	332		0.16	0.92 (−4, 1.4)
Sensorimotor dexterity		263	−0.20	1.0 (−4.0, 0.1)	345		0.07	0.37 (−4, 0.12)
Emotion processing		261	−0.88	1.4 (−4.0, 1.9)	348		0.13	0.86 (−3.9, 1.9)

Abbreviations: CNB, Computerized Neurocognitive Battery; CPT-IP, Continuous Performance Test, Identical Pairs version; CVLT, California Verbal Learning Test; DS-CPT, Degraded Stimulus Continuous Performance Test; LNS, Letter-Number Span; PPI, prepulse inhibition.

a Proportion correct out of a maximum of 60 trials.

b Prepulse inhibition using 60 msec pulses.

100 × [1 − (magnitude of startle to pulse preceded by prepulse/magnitude of startle to pulse without a preceding prepulse)].

c Overall signal/noise discrimination (d′).

d Three-digit d′.

e Four-digit d′.

f After each sequence, the participant is asked to recall the numbers and letters in the same exact order, with no reordering of the stimuli. The number of digits and letters increases by one on each trial, from one up to a maximum length of 8 stimuli. Three sequences of the same length are presented during each trial. The test is discontinued when the subject fails three consecutive sequences of the same length. The score is the total number of correctly recalled sequences.

g After each sequence, the participant is asked to repeat the numbers in ascending order first and then the letters in alphabetical order.

h Trials 1–5 Free Recall Correct.

i Total semantic clustering scores on trials 1–5.

j The number of correct responses standardized to a z-score based on the COGS community control subjects.

**Table 3:** Significant terms involving paternal age at birth in linear and linear mixed-effects models for endophenotype performance.

	Schizophrenia subjects only		Schizophrenia subjects and unaffected siblings combined	
Endophenotype	Significant term^a^ (slope^b^, *P* value^c^^,^ d, 95% CI)	*R^2^*	Significant term^a^ (slope^b^, *P* value^d^^,^ e, 95% CI)	*R^2^*
CPT-IP 4-digit	Paternal age (slope = 0.19, *P* = 0.03, 95% CI = [0.01, 0.36])	0.11	Paternal age (slope = 0.20, *P* = 0.02, 95% CI = [0.03, 0.37])	0.41
Verbal memory	Paternal age by Multiplex status (Simplex slope = −0.07, 95% CI = [−0.42, 0.29]; Multiplex slope = −1.0, 95% CI = [−1.9, −0.15]; *P* for difference in slopes = 0.03)	0.13	Paternal age by Multiplex status (Simplex slope = −0.05, 95% CI = [−0.34, 0.25]; Multiplex slope = −0.89, 95% CI = [−1.5, −0.32]; *P* for difference in slopes = 0.005)	0.30
			Paternal age by Gender (Male slope = −0.05, 95% CI = [−0.34, 0.25]; Female slope = −0.43, 95% CI = [−0.83, −0.03]; *P* for difference in slopes = 0.049)	

Abbreviations: CI, confidence interval; CPT-IP, Continuous Performance Test, Identical Pairs version.

a Only terms involving paternal age at subject birth with an associated *P* value <0.05 are reported, and only endophenotypes with such terms are reported.

b Slope and confidence intervals are in units of a 10-year increase in paternal age at birth. A *positive* slope indicates that subjects with older fathers perform *better* on the endophenotype.

c *P* values are based on linear models with effects for paternal age, paternal age–by-gender, and paternal age–by–multiplex status, with subject age, test site, subject gender, and parental education as covariates.

d After adjusting for multiple comparisons accounting for the 16 endophenotypes, none of the results are significant at an overall Type I error level of 5%.

e *P* values are based on linear mixed-effects models with effects for paternal age, paternal age–by-gender, and paternal age–by–multiplex status, with group (proband versus sibling), subject age, test site, subject gender, parental education, and all second-order interactions involving group as covariates. Family membership served as a random effect to account for the relatedness of observations among family members.

Of the 16 endophenotypes considered, only the 4-digit CPT-IP (CPT-IP 4-digit) and CNB verbal memory test showed nominally significant associations with paternal age. CPT-IP 4-digit showed a positive association with paternal age (i.e., subjects with older fathers performed better), and the CNB verbal memory test showed an interaction effect between paternal age and multiplex status (test scores were more negatively associated with paternal age for multiplex subjects than simplex subjects). In the analysis of both probands and unaffected siblings, the verbal memory test also showed an interaction effect between paternal age and gender (test scores were more negatively associated with paternal age for females than males). After adjusting for multiple comparisons accounting for the 16 endophenotypes, none of the results remained statistically significant (Table 3 shows only results involving paternal age that are nominally significant before adjusting for multiple comparisons; Table S1 shows results for all terms involving paternal age for all endophenotypes, regardless of significance).

For the sensitivity analyses, when paternal age at birth was treated as continuous and interaction terms were omitted from the models, only the CPT-IP 4-digit and the California Verbal Learning Test semantic clustering (CVLT semantic) showed significant associations with paternal age. CPT-IP 4-digit again showed a positive association with paternal age and CVLT semantic showed a negative association. After adjusting for multiple comparisons accounting for the 16 endophenotypes, only the effect of paternal age on CPT-IP 4-digit in the probands-only analysis remained statistically significant. After adjusting for multiple comparisons accounting for the 16 endophenotypes and two models (probands-only versus probands plus unaffected siblings), none of the results remained statistically significant.

When paternal age at birth was treated as categorical (<40 versus ≥40) and interaction terms were omitted from the models, CPT-IP 4-digit again showed a positive association with paternal age, but this association was not significant after adjusting for multiple comparisons accounting for the 16 endophenotypes. When paternal age at birth was treated as categorical (<40 versus ≥40) and interaction terms were included in the models, CPT-IP 3-digit, CPT-IP 4-digit, and CNB spatial ability all showed positive associations with paternal age. Also, similar to when paternal age was treated as continuous, in the analysis of both probands and unaffected siblings, CNB verbal memory showed an interaction effect between paternal age and multiplex status (test scores were more negatively associated with paternal age for multiplex subjects than simplex subjects). CNB sensorimotor dexterity showed the same kind of paternal age–by–multiplex status interaction effect, and CNB abstraction/mental flexibility showed an interaction effect between paternal age and group (scores were positively associated with paternal age for probands and negatively associated with paternal age for unaffected siblings). After adjusting for multiple comparisons accounting for the 16 endophenotypes, only two effects remained statistically significant: the effect of paternal age on spatial ability, for probands only, and the paternal age–by–multiplex status interaction, for probands and their unaffected siblings. After adjusting for multiple comparisons accounting for the 16 endophenotypes and two models, none of the results remained statistically significant.

## Discussion

Our overall findings do not suggest that advanced paternal age at birth negatively affects endophenotypes associated with schizophrenia; rather, our findings imply that factors other than paternal age at birth are operating to influence performance on these neurocognitive and neurophysiologic measures.

The current study operated under the initial hypothesis that advanced paternal age may serve as a proxy for the presence of genetic variations that may increase the risk of developing schizophrenia and may also potentially increase schizophrenia-associated endophenotypic deficit levels. Some investigators have hypothesized that the association between the risk for sporadic schizophrenia and advanced paternal age can be attributed to *de novo* mutations in paternal germ lines [11]. Specifically, given the increased rate of mitotic cell division in the sperm cells relative to the oocyte, the possibility of error in gene replication greatly increases along with advanced paternal age. Indeed, advanced paternal age has been linked with human and rodent sex chromosomal aneuploidies as well as a fourfold increase in structural chromosomal abnormalities (i.e., breaks and/or rearrangements) and other DNA damage in human and mouse sperm [45]. Thus, molecular mechanisms associated with aging males could explain the relationship between advanced paternal age and the development of major psychiatric disorders such as schizophrenia.

However, in the current study, paternal age at birth played little to no role in influencing performance on endophenotypic measures. For example, although we found an association between paternal age at birth and performance on the CPT-IP 4-digit, this association ran counter to our hypothesis in that our subjects with older fathers actually performed better on this measure. In the analyses that combined the probands and unaffected siblings, we found a significant paternal age–by–multiplex status interaction with the verbal memory component of the CNB such that test scores in multiplex subjects showed a more negative association with paternal age than in simplex subjects. However, after adjustment for multiple comparisons, none of these associations remained statistically significant, suggesting that these findings may be due primarily to Type I error.

As for clinical symptoms, there was no difference in negative symptoms (as measured by the SANS) nor age of onset between the probands with younger fathers and the probands with older fathers. This finding contrasts with recent research demonstrating a relationship between advanced paternal age at birth and positive symptoms as measured by the PANSS [20]. It is possible that the differences between our findings and the findings in the study by Rosenfield and colleagues [20] could be explained by differences in our study design; for example, our study included probands who were on antipsychotic medications (their symptoms may have therefore been less severe), whereas the study by Rosenfield and colleagues only included unmedicated subjects. Furthermore, our study used a family-based design rather than a case-control design, and this difference in recruitment methodology may have contributed to our nonsignificant findings.

Our study has some limitations. First, as mentioned in our comparison with the study by Rosenfield and colleagues, the severity of schizophrenia symptoms might be more evident when subjects are not on antipsychotics, and thus our inclusion of such subjects may make our results difficult to compare to studies that excluded subjects on antipsychotics. Second, subjects who were missing data for paternal age at birth tended to display poorer clinical outcomes than subjects with paternal age data available. If these subjects tended to have higher (or lower) paternal age at birth than the subjects with paternal age data available, this could potentially bias our results. And third, our study had a relatively high number of subjects with missing endophenotype data, and if these missing values were not missing at random, there is a potential for bias.

In summary, findings from our study do not suggest that advanced paternal age has a substantial effect on the endophenotypic deficits in schizophrenia probands or their unaffected siblings, nor on the severity of clinical symptoms or the age of onset of schizophrenia. The strength of this study is that its large, clinically well-characterized family-based sample enabled a thorough investigation of these associations. Another strength of our study is that we collected endophenotypic data from unaffected siblings in families with schizophrenia (not unrelated controls), which allowed us to make direct comparisons between affected and unaffected individuals who had the same fathers. Although the mechanisms by which advanced paternal age increases the risk of developing schizophrenia remain unknown, they do not appear to play a substantial role in the presence of endophenotypic deficits in schizophrenia. Additional studies are necessary to increase our understanding of the ways in which factors associated with advanced paternal age may adversely affect the developing brain.

## Supporting Information

Table S1 Terms involving paternal age at birth in linear and linear mixed-effects models for endophenotype performance. Abbreviations: CI, confidence interval; CPT-IP, Continuous Performance Test, Identical Pairs version; LNS, Letter-Number Span; CVLT, California Verbal Learning Test. ^a^ Only terms involving paternal age at subject birth are reported. ^b^ Slope and confidence intervals are in units of a 10-year increase in paternal age at birth. A *positive* slope indicates that subjects with older fathers perform *better* on the endophenotype. ^c^ *P* values are based on linear models with effects for paternal age, paternal age–by-gender, and paternal age–by–multiplex status, with subject age, test site, subject gender, and parental education as covariates. ^d^ After adjusting for multiple comparisons accounting for the 16 endophenotypes, none of the results are significant at an overall Type I error level of 5%. ^e^ *P* values are based on linear mixed-effects models with effects for paternal age, paternal age–by-gender, and paternal age–by–multiplex status, with group (proband versus sibling), subject age, test site, subject gender, parental education, and all second-order interactions involving group as covariates. Family membership served as a random effect to account for the relatedness of observations among family members.
(DOCX)
