# Supplementary material for: Is There an Association between Advanced Paternal Age and Endophenotype Deficit Levels in Schizophrenia?
Source: PLoS One. 2014 Feb 11;9(2):e88379. doi: 10.1371/journal.pone.0088379 (PMC3921166; doi:10.1371/journal.pone.0088379)
Supplement: Table S1 — Terms involving paternal age at birth in linear and linear mixed-effects models for endophenotype performance. Abbreviations: CI, confidence interval; CPT-IP, Continuous Performance Test, Identical Pairs version; LNS, Letter-Number Span; CVLT, California Verbal Learning Test. a Only terms involving paternal age at subject birth are reported. b Slope and confidence intervals are in units of a 10-year increase in paternal age at birth. A positive slope indicates that subjects with older fathers perform better on the endophenotype. c P values are based on linear models with effects for paternal age, paternal age–by-gender, and paternal age–by–multiplex status, with subject age, test site, subject gender, and parental education as covariates. d After adjusting for multiple comparisons accounting for the 16 endophenotypes, none of the results are significant at an overall Type I error level of 5%. e P values are based on linear mixed-effects models with effects for paternal age, paternal age–by-gender, and paternal age–by–multiplex status, with group (proband versus sibling), subject age, test site, subject gender, parental education, and all second-order interactions involving group as covariates. Family membership served as a random effect to account for the relatedness of observations among family members. (DOCX) [file pone.0088379.s001.docx]

**Table S1**

Terms involving paternal age at birth in linear and linear mixed-effects models for endophenotype performance.

|  | Schizophrenia subjects only | |  | Schizophrenia subjects and unaffected siblings combined | |
| --- | --- | --- | --- | --- | --- |
| Endophenotype | Term^a^ (slope^b^, *P* value^c,d^, 95% CI) | *R^2^* |  | Term^a^ (slope^b^, *P* value^d,e^, 95% CI) | *R^2^* |
| Antisaccade proportion correct | Paternal age (slope=0.0005, *P*=0.99, 95% CI=[-0.07, 0.07]) | 0.15 |  | Paternal age (slope=-0.008, *P*=0.76, 95% CI=[-0.06, 0.05]) | 0.56 |
|  | Paternal age by Multiplex status (Multiplex slope - Simplex slope=-0.02, *P*=0.84, 95% CI=[-0.16, 0.13]) |  |  | Paternal age by Multiplex status (Multiplex slope - Simplex slope=-0.05, *P*=0.33, 95% CI=[-0.14, 0.05]) |  |
|  | Paternal age by Gender (Female slope - Male slope=-0.07, *P*=0.26, 95% CI=[-0.17, 0.05]) |  |  | Paternal age by Gender (Female slope - Male slope=0.001, *P*=0.96, 95% CI=[-0.06, 0.06]) |  |
|  |  |  |  | Paternal age by group (Proband slope - Sibling slope=0.003, *P*=0.91, 95% CI=[-0.05, 0.06]) |  |
| Prepulse inhibition (PPI) | Paternal age (slope=-1.7, *P*=0.69, 95% CI=[-9.9, 6.6]) | 0.13 |  | Paternal age (slope=-3.2, *P*=0.38, 95% CI=[-10, 3.9]) | 0.29 |
|  | Paternal age by Multiplex status (Multiplex slope - Simplex slope=-0.9, *P*=0.91, 95% CI=[-17, 15]) |  |  | Paternal age by Multiplex status (Multiplex slope - Simplex slope=2.7, *P*=0.62, 95% CI=[-8.1, 14]) |  |
|  | Paternal age by Gender (Female slope - Male slope=-1.5, *P*=0.83, 95% CI=[-15, 12]) |  |  | Paternal age by Gender (Female slope - Male slope=0.48, *P*=0.91, 95% CI=[-7.6, 8.5]) |  |
|  |  |  |  | Paternal age by group (Proband slope - Sibling slope=-0.61, *P*=0.88, 95% CI=[-8.9, 7.7]) |  |
| CPT-IP 3-digit | Paternal age (slope=0.07, *P*=0.54, 95% CI=[-0.14, 0.27]) | 0.09 |  | Paternal age (slope=0.09, *P*=0.34, 95% CI=[-0.09, 0.26]) | 0.50 |
|  | Paternal age by Multiplex status (Multiplex slope - Simplex slope=0.23, *P*=0.41, 95% CI=[-0.32, 0.79]) |  |  | Paternal age by Multiplex status (Multiplex slope - Simplex slope=0.19, *P*=0.27, 95% CI=[-0.15, 0.52]) |  |
|  | Paternal age by Gender (Female slope - Male slope=0.20, *P*=0.32, 95% CI=[-0.20, 0.61]) |  |  | Paternal age by Gender (Female slope - Male slope=0.14, *P*=0.18, 95% CI=[-0.07, 0.35]) |  |
|  |  |  |  | Paternal age by group (Proband slope - Sibling slope=-0.19, *P*=0.08, 95% CI=[-0.40, 0.02]) |  |
| CPT-IP 4-digit | Paternal age (slope=0.19, *P*=0.03, 95% CI=[0.01, 0.36]) | 0.11 |  | Paternal age (slope=0.20, *P*=0.02, 95% CI=[0.03, 0.37]) | 0.41 |
|  | Paternal age by Multiplex status (Multiplex slope - Simplex slope=-0.21, *P*=0.36, 95% CI=[-0.67, 0.24]) |  |  | Paternal age by Multiplex status (Multiplex slope - Simplex slope=0.09, *P*=0.56, 95% CI=[-0.22, 0.41]) |  |
|  | Paternal age by Gender (Female slope - Male slope=0.25, *P*=0.14, 95% CI=[-0.08, 0.59]) |  |  | Paternal age by Gender (Female slope - Male slope=0.02, *P*=0.88, 95% CI=[-0.19, 0.22]) |  |
|  |  |  |  | Paternal age by group (Proband slope - Sibling slope=-0.18, *P*=0.09, 95% CI=[-0.39, 0.03]) |  |
| LNS Forward | Paternal age (slope=0.46, *P*=0.17, 95% CI=[-0.19, 1.1]) | 0.10 |  | Paternal age (slope=0.32, *P*=0.29, 95% CI=[-0.28, 0.91]) | 0.49 |
|  | Paternal age by Multiplex status (Multiplex slope - Simplex slope=-0.11, *P*=0.89, 95% CI=[-1.7, 1.4]) |  |  | Paternal age by Multiplex status (Multiplex slope - Simplex slope=0.39, *P*=0.50, 95% CI=[-0.74, 1.5]) |  |
|  | Paternal age by Gender (Female slope - Male slope=-0.11, *P*=0.86, 95% CI=[-1.3, 1.1]) |  |  | Paternal age by Gender (Female slope - Male slope=0.14, *P*=0.69, 95% CI=[-0.55, 0.84]) |  |
|  |  |  |  | Paternal age by group (Proband slope - Sibling slope=-0.51, *P*=0.14, 95% CI=[-1.2, 0.17]) |  |
| LNS Reordered | Paternal age (slope=0.55, *P*=0.10, 95% CI=[-0.10, 1.2]) | 0.15 |  | Paternal age (slope=0.32, *P*=0.25, 95% CI=[-0.23, 0.87]) | 0.51 |
|  | Paternal age by Multiplex status (Multiplex slope - Simplex slope=-0.45, *P*=0.57, 95% CI=[-2.0, 1.1]) |  |  | Paternal age by Multiplex status (Multiplex slope - Simplex slope=0.17, *P*=0.75, 95% CI=[-0.88, 1.2]) |  |
|  | Paternal age by Gender (Female slope - Male slope=-0.82, *P*=0.17, 95% CI=[-2.0, 0.35]) |  |  | Paternal age by Gender (Female slope - Male slope=-0.41, *P*=0.21, 95% CI=[-1.1, 0.24]) |  |
|  |  |  |  | Paternal age by group (Proband slope – Sibling slope=-0.11, *P*=0.73, 95% CI=[-0.75, 0.52]) |  |
| CVLT total | Paternal age (slope=-2.3, *P*=0.09, 95% CI=[-5.0, 0.34]) | 0.28 |  | Paternal age (slope=-1.9, *P*=0.08, 95% CI=[-4.0, 0.23]) | 0.61 |
|  | Paternal age by Multiplex status (Multiplex slope - Simplex slope=-1.5, *P*=0.63, 95% CI=[-7.7, 4.7]) |  |  | Paternal age by Multiplex status (Multiplex slope - Simplex slope=-0.81, *P*=0.68, 95% CI=[-4.7, 3.1]) |  |
|  | Paternal age by Gender (Female slope - Male slope=2.0, *P*=0.39, 95% CI=[-2.6, 6.7]) |  |  | Paternal age by Gender (Female slope - Male slope=0.46, *P*=0.72, 95% CI=[-2.9, 2.0]) |  |
|  |  |  |  | Paternal age by group (Proband slope - Sibling slope=2.0, *P*=0.11, 95% CI=[-0.47, 4.4]) |  |
| CVLT semantic | Paternal age (slope=-0.33, *P*=0.07, 95% CI=[-0.69, 0.03]) | 0.07 |  | Paternal age (slope=-0.30, *P*=0.15, 95% CI=[-0.71, 0.11]) | 0.37 |
|  | Paternal age by Multiplex status (Multiplex slope - Simplex slope=0.21, *P*=0.63, 95% CI=[-0.63, 1.0]) |  |  | Paternal age by Multiplex status (Multiplex slope - Simplex slope=-0.23, *P*=0.54, 95% CI=[-0.95, 0.50]) |  |
|  | Paternal age by Gender (Female slope - Male slope=-0.07, *P*=0.84, 95% CI=[-0.69, 0.56]) |  |  | Paternal age by Gender (Female slope - Male slope=0.02, *P*=0.93, 95% CI=[-0.50, 0.46]) |  |
|  |  |  |  | Paternal age by group (Proband slope - Sibling slope=0.46, *P*=0.06, 95% CI=[-0.03, 0.94]) |  |
| Abstraction and mental flexibility | Paternal age (slope=0.17, *P*=0.23, 95% CI=[-0.11, 0.45]) | 0.13 |  | Paternal age (slope=0.09, *P*=0.41, 95% CI=[-0.13, 0.31]) | 0.39 |
|  | Paternal age by Multiplex status (Multiplex slope - Simplex slope=-0.18, *P*=0.58, 95% CI=[-0.84, 0.47]) |  |  | Paternal age by Multiplex status (Multiplex slope - Simplex slope=-0.10, *P*=0.64, 95% CI=[-0.50, 0.31]) |  |
|  | Paternal age by Gender (Female slope - Male slope=-0.28, *P*=0.27, 95% CI=[-0.78, 0.22]) |  |  | Paternal age by Gender (Female slope - Male slope=0.01, *P*=0.93, 95% CI=[-0.25, 0.27]) |  |
|  |  |  |  | Paternal age by group (Proband slope - Sibling slope=-0.13, *P*=0.32, 95% CI=[-0.40, 0.13]) |  |
| Verbal memory | Paternal age (slope=-0.07, *P*=0.72, 95% CI=[-0.42, 0.29]) | 0.13 |  | Paternal age (slope=-0.05, *P*=0.74, 95% CI=[-0.34, 0.25]) | 0.30 |
|  | Paternal age by Multiplex status (Multiplex slope - Simplex slope=-0.98, *P*=0.03, 95% CI=[-1.9, -0.08]) |  |  | Paternal age by Multiplex status (Multiplex slope - Simplex slope=-0.84, *P*=0.005, 95% CI=[-1.4, -0.26]) |  |
|  | Paternal age by Gender (Female slope - Male slope=-0.26, *P*=0.48, 95% CI=[-0.97, 0.46]) |  |  | Paternal age by Gender (Female slope - Male slope=-0.38, *P*=0.049, 95% CI=[-0.76, -0.001]) |  |
|  |  |  |  | Paternal age by group (Proband slope - Sibling slope=0.39, *P*=0.051, 95% CI=[-0.001, 0.78]) |  |
| Face memory | Paternal age (slope=-0.13, *P*=0.35, 95% CI=[-0.41, 0.15]) | 0.09 |  | Paternal age (slope=-0.11, *P*=0.30, 95% CI=[-0.33, 0.10]) | 0.49 |
|  | Paternal age by Multiplex status (Multiplex slope - Simplex slope=0.10, *P*=0.76, 95% CI=[-0.56, 0.77]) |  |  | Paternal age by Multiplex status (Multiplex slope - Simplex slope=-0.05, *P*=0.83, 95% CI=[-0.46, 0.37]) |  |
|  | Paternal age by Gender (Female slope - Male slope=-0.02, *P*=0.94, 95% CI=[-0.52, 0.48]) |  |  | Paternal age by Gender (Female slope - Male slope=0.007, *P*=0.95, 95% CI=[-0.25, 0.27]) |  |
|  |  |  |  | Paternal age by group (Proband slope - Sibling slope=0.06, *P*=0.65, 95% CI=[-0.20, 0.31]) |  |
| Spatial memory | Paternal age (slope=-0.17, *P*=0.17, 95% CI=[-0.42, 0.08]) | 0.15 |  | Paternal age (slope=-0.16, *P*=0.12, 95% CI=[-0.36, 0.04]) | 0.55 |
|  | Paternal age by Multiplex status (Multiplex slope - Simplex slope=-0.10, *P*=0.72, 95% CI=[-0.68, 0.47]) |  |  | Paternal age by Multiplex status (Multiplex slope - Simplex slope=0.19, *P*=0.33, 95% CI=[-0.19, 0.58]) |  |
|  | Paternal age by Gender (Female slope - Male slope=0.22, *P*=0.33, 95% CI=[-0.22, 0.66]) |  |  | Paternal age by Gender (Female slope - Male slope=0.05, *P*=0.65, 95% CI=[-0.18, 0.29]) |  |
|  |  |  |  | Paternal age by group (Proband slope - Sibling slope=0.11, *P*=0.36, 95% CI=[-0.12, 0.34]) |  |
| Spatial processing | Paternal age (slope=0.20, *P*=0.10, 95% CI=[-0.04, 0.44]) | 0.25 |  | Paternal age (slope=0.13, *P*=0.19, 95% CI=[-0.06, 0.33]) | 0.56 |
|  | Paternal age by Multiplex status (Multiplex slope - Simplex slope=-0.43, *P*=0.17, 95% CI=[-1.1, 0.17]) |  |  | Paternal age by Multiplex status (Multiplex slope - Simplex slope=-0.12, *P*=0.60, 95% CI=[-0.54, 0.31]) |  |
|  | Paternal age by Gender (Female slope - Male slope=-0.10, *P*=0.66, 95% CI=[-0.54, 0.34]) |  |  | Paternal age by Gender (Female slope - Male slope=-0.03, *P*=0.82, 95% CI=[-0.26, 0.21]) |  |
|  |  |  |  | Paternal age by group (Proband slope - Sibling slope=-0.08, *P*=0.47, 95% CI=[-0.32, 0.15]) |  |
| Sensorimotor dexterity | Paternal age (slope=0.13, *P*=0.27, 95% CI=[-0.10, 0.35]) | 0.17 |  | Paternal age (slope=0.13, *P*=0.10, 95% CI=[-0.02, 0.27]) | 0.24 |
|  | Paternal age by Multiplex status (Multiplex slope - Simplex slope=-0.24, *P*=0.40, 95% CI=[-0.79, 0.32]) |  |  | Paternal age by Multiplex status (Multiplex slope - Simplex slope=-0.18, *P*=0.18, 95% CI=[-0.44, 0.09]) |  |
|  | Paternal age by Gender (Female slope - Male slope=0.05, *P*=0.80, 95% CI=[-0.35, 0.46]) |  |  | Paternal age by Gender (Female slope - Male slope=0.03, *P*=0.75, 95% CI=[-0.15, 0.21]) |  |
|  |  |  |  | Paternal age by group (Proband slope - Sibling slope=-0.14, *P*=0.14, 95% CI=[-0.32, 0.05]) |  |
| Emotion processing | Paternal age (slope=0.04, *P*=0.81, 95% CI=[-0.29, 0.37]) | 0.04 |  | Paternal age (slope=0.06, *P*=0.65, 95% CI=[-0.19, 0.30]) | 0.37 |
|  | Paternal age by Multiplex status (Multiplex slope - Simplex slope=-0.55, *P*=0.17, 95% CI=[-1.3, 0.23]) |  |  | Paternal age by Multiplex status (Multiplex slope - Simplex slope=-0.27, *P*=0.23, 95% CI=[-0.72, 0.18]) |  |
|  | Paternal age by Gender (Female slope - Male slope=0.08, *P*=0.78, 95% CI=[-0.51, 0.68]) |  |  | Paternal age by Gender (Female slope - Male slope=-0.10, *P*=0.48, 95% CI=[-0.40, 0.19]) |  |
|  |  |  |  | Paternal age by group (Proband slope - Sibling slope=0.08, *P*=0.59, 95% CI=[-0.22, 0.38]) |  |

Abbreviations: CI, confidence interval; CPT-IP, Continuous Performance Test, Identical Pairs version; LNS, Letter-Number Span; CVLT, California Verbal Learning Test.

^a^ Only terms involving paternal age at subject birth are reported.
^b^ Slope and confidence intervals are in units of a 10-year increase in paternal age at birth. A *positive* slope indicates that subjects with older fathers perform *better* on the endophenotype. ^c^ *P* values are based on linear models with effects for paternal age, paternal age–by-gender, and paternal age–by–multiplex status, with subject age, test site, subject gender, and parental education as covariates.
^d^ After adjusting for multiple comparisons accounting for the 16 endophenotypes, none of the results are significant at an overall Type I error level of 5%.

^e^ *P* values are based on linear mixed-effects models with effects for paternal age, paternal age–by-gender, and paternal age–by–multiplex status, with group (proband versus sibling), subject age, test site, subject gender, parental education, and all second-order interactions involving group as covariates. Family membership served as a random effect to account for the relatedness of observations among family members.
